# The influence of myofascial release on pain and selected indicators of flat foot in adults: a controlled randomized trial

**DOI:** 10.1038/s41598-022-05401-w

**Published:** 2022-01-26

**Authors:** Aneta Bac, Sabina Kaczor, Szymon Pasiut, Anna Ścisłowska-Czarnecka, Agnieszka Jankowicz-Szymańska, Katarzyna Filar-Mierzwa

**Affiliations:** 1 Faculty of Motor Rehabilitation, The Bronisław Czech, University of Physical Education, al. Jana Pawła II 78, 31-571 Krakow, Poland; 2 Faculty of Health Science, University of Applied Science in Tarnow, Tarnów, Poland

**Keywords:** Pain management, Rehabilitation

## Abstract

Flat foot pain is a common complaint that requires therapeutic intervention. Currently, myofascial release techniques are often used in the therapy of musculoskeletal disorders. A group of 60 people suffering from flat feet with associated pain. Patients were assigned to four groups (15 people each): MF—myofascial release, E—the exercise program, MFE—myofascial release and the exercise program, C—no intervention. The rehabilitation program lasted 4 weeks. The NRS scale was used to examine pain intensity and FreeMed ground reaction force platform was used to examine selected static and dynamic foot indicators. Statistically significant pain reduction was obtained in all research. A static test of foot load distribution produced statistically significant changes only for selected indicators. In the dynamic test, statistically significant changes were observed for selected indicators, only in the groups subjected to therapeutic intervention. Most such changes were observed in the MF group. In the dynamic test which assessed the support phase of the foot, statistically significant changes were observed only for selected subphases. Most such changes were observed in the MFE group. Both exercise and exercise combined with myofascial release techniques, and especially myofascial release techniques alone, significantly reduce pain in a flat foot. This study shows a limited influence of both exercises and myofascial release techniques on selected static and dynamic indicators of a flat foot.

## Introduction

Pain in the foot is a common and complex problem. The risk of the occurrence of pain is directly related to the shape and functioning of the feet^1^. It is assumed that deviations from the norm in terms of arching of the feet affect the walking pattern, which results in excessive overloading of bones and soft tissues. Structural changes have a negative impact on functional efficiency, and the pain that appears in the course of these pathologies, impairment of the range of motion, or weakening of muscle strength trigger compensatory mechanisms that aggravate the dysfunctions^1–3^. Foot pain is an important problem as it can also have a negative impact on daily activities, fitness and quality of life, and may increase the risk of falling^4,5^.

The problem of flat foot, its etiology, diagnostics and treatment is often addressed by researchers. A considerable number of the publication concentrate on the population of children and adolescents^6–8^; however, it should be remembered that this disorder also affects adults^7,9–12^. A symptom of flat foot, in addition to the lowered longitudinal arch of the foot, is also pain, which is the main reason for medical and physiotherapeutic consultations^1^. In addition, there are reports in the literature suggesting that decreased arching may cause adverse changes in foot loading parameters (static and dynamic) and in the biomechanics of gait, thus contributing to overloading of soft tissues and bones^1,13,14^.

In physiotherapeutic practice, manual therapy is an increasingly popular method of working with a patient with pain. Manual therapy used for flat foot pain includes both soft tissue methods and joint mobilization. In clinical practice, these two techniques are often combined to achieve a faster and better therapeutic outcome^15^. In recent years, soft tissue therapy, including myofascial release, trigger point therapy, and soft tissue mobilization, has gained increasing use, despite the fact that there are reports in the available literature showing that the role of these techniques is not fully explained^16,17^ or their influence is limited^18,19^.

Among the above techniques, myofascial release techniques, which are used in the treatment of various dysfunctions of the musculoskeletal system, such as pain of muscular origin or limitations of the range of motion in joints, are becoming more widespread and accepted^11,20–23^; however, a very small percentage of these reports relate to the foot, and most focus only on plantar fasciitis^24,25^.

The available literature, however, lacks articles on myofascial release dedicated to flat foot with pain and on the impact of such therapy on ground foot pressure in static and dynamic conditions. To the best of our knowledge, our research is the first to analyze the effectiveness of myofascial release as an individual (separate) therapy and as a therapy that is combined with exercises as compared with an exercise program and the control group in adults with flat foot pain.

## Material and methods

### Participants

**Figure 1:**
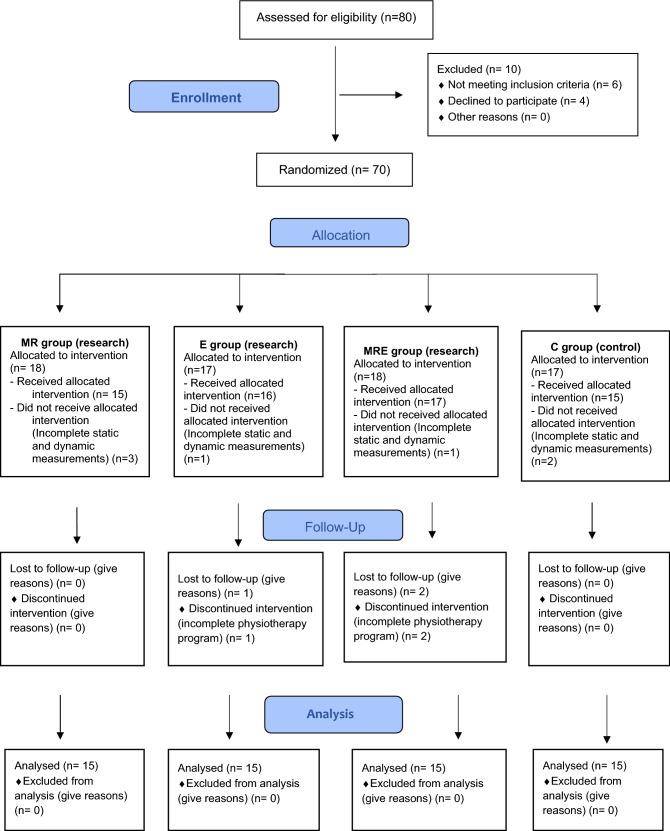
Consort diagram.

This is the pre post treatment randomized controlled trial. A group of 60 people (randomly divided into 4 groups) took part in the research project (see Fig. 1). All subjects reported foot pain and had flat feet. At the end there were four groups, 15 patients each in the age of 20–49.

The following inclusion criteria were assumed: flat foot (not rigid); age between 20 and 50; pain in the foot; no injuries affecting the efficiency of the lower limbs in the last 6 months; no neurological, metabolic, rheumatic or orthopedic diseases; no contraindications to therapy; written consent for the study.

Qualification was based on the simple randomization (coin toss) performed by the main author. The subjects were assigned to four groups:

- group MF (15 people), subjected to a 4-week rehabilitation program which covered only myofascial release
- group E (15 people), subjected to a 4-week exercise-based rehabilitation program performed daily, throughout the duration of the project
- group MFE (15 people), subjected to a 4-week rehabilitation program which involved myofascial release and a set of exercises performed daily
- group C (15 people), control group (no intervention).

Until the final preparation of the database, the main author was the only person who knew which group each researched person was assigned to. The therapy was performed by other therapists, and the examinations were performed by another member of the therapeutic team.

The study was approved by the Bioethics Committee at the Regional Medical Chamber in Krakow (No. 94/KBL/OIL/2016).

Measurements were carried out at the University of Physical Education in Faculty of Motor Rehabilitation, in collaboration with Medical Centre Liszki. The research was conducted in 2017–2019. The study was conducted in accordance with the Code of Ethics of the World Medical Association (Declaration of Helsinki 1964). The informed consent was obtained for each patient.

This study was registered in Australian and New Zealand Clinical Trials Registry. Registration number: ACTRN12617000257369 (date registered: 20/02/2017). Patients’ written consent was obtained, and the rights of subjects were protected.

This pre post treatment trial was reported according to the recommendations of the Consolidated Standards of Reporting Trials (CONSORT) statement^26^.

### Assessment tools

In all participants (control and researched groups) the clinical examination was performer twice, before and at the end of rehabilitation protocol, excluding podoscopic examination which was performed once before the onset of the study and was used to include or exclude the patients. Following assessments were performed:

- Podoscopic examination (to qualify the patients for research): during podoscopic examination both feet were measured simultaneously. To evaluate the degree of the flattening of the longitudinal arch of the foot based on a scanner image, the Clarke angle was calculated as the angle between the tangent of the medial margin of the foot print and the line connecting the point of largest recess and the contact of the medial tangent with the border of the forefoot^27^. Podoscopic examination is commonly used to diagnose various foot pathologies, both in children and adults^28,29^.
- NRS scale (to evaluate the pain intensity): A 10-point VAS scale was used to measure pain intensity, with 0 indicating no pain and 10 the highest possible pain. The NRS scale has for many years been considered one of the most reliable tools for pain intensity measurements^30,31^.
- FreeMed ground reaction force platform (to calculate foot load distribution): during the *static tests* on the FreeMed ground reaction force platform the foot load distribution was calculated. The static test was performed in a free standing position, with arms hanging freely along the torso and feet parallel to each other, slightly apart, barefoot. The first measurement was a test measurement, the second one was the main measurement. As a result of the study, the percentage load distribution was divided into foot regions (the foot was divided into 6 regions: medial forefoot, lateral forefoot, medial midfoot, lateral midfoot, medial hindfoot, and lateral hindfoot). During *dynamic tests* on the FreeMed ground reaction force platform selected foot indicators during walking were calculated. During the dynamic test, the patient was asked to walk on the measurement path at their own pace 12 times. Before the actual measurement began, the patient walked on the path five times to prepare for the study. The results of the measurements included: the size of the load surface (cm^2^); stance time (ms); the value of the vertical component of the ground reaction force (N): first and second maximum (vGRF F1, vGRF F3) and the first minimum (vGRF F2); selected indicators in the support phase sub-phases (IC—Initial Contact, LR—Loading Response, MidSt—Midstance, TSt—Terminal Stance): duration (ms) and percentage of maximum load (%).This platform is commonly used to measure static and dynamic foot indicators in patients of different age and with various dysfunctions^32–35^.

### Intervention

Both myofascial release and exercises was performed by the same therapist in all patients.

- Myofascial release: the subjects from the MF group and the MFE group participated in myofascial release sessions. Meetings were held for 4 weeks, twice a week, and lasted 40 min (20 min for each lower limb). The purpose of this techniques was to reduce a pain and increase muscle mobility and flexibility. The so-called direct techniques of myofascial release were used, among them lengthening of peroneal muscles, lifting of the plantar flexors (the gastrocnemius, and the soleus muscle), working to elongate the gastrocnemius and soleus calf muscles, working on the Achilles tendon, working on the tissues around the heel, working on plantar fascia, working on the furrows (the peroneal muscle/the soleus muscle, the gastrocnemius/the soleus muscle, the Achilles tendon/tendon crossing).
- Exercises: subjects from the MFE group and group E performed a set of exercises under the guidance of a physiotherapist daily (from Monday to Friday) for 4 weeks. The purpose of this exercises was to reduce a pain, increase muscle strength and flexibility. The series of exercises comprised seven exercises, divided into two parts. The first part involved stretching selected leg muscles: the gastrocnemius (standing position, hold position time—20 s, number of repetitions—5), the soleus (standing position, hold position time—20 s., number of repetitions—5), the peroneal muscles (standing position, hold position time—20 s., number of repetitions—5) and the plantar aponeurosis (standing position, with roller, number of repetitions—10 and siting position, hold position time—20 s., number of repetitions—5). The second part consisted of exercises strengthening the tibial posterior muscle (standing position, hold position time—10 s., number of repetitions—10), the flexor muscles of the toes (standing position, hold position time—10 s., number of repetitions—10), and the short internal muscles of the foot on the plantar side (sitting position, hold position time—10 s, number of repetitions—10). The duration of therapy was 30 min a day.

### Statistical methods

Statistical analysis of the gathered data was analyzed using Statistica 10.0 (StatSoft). The following parameters were used: mean average, median, minimal and maximal values and standard deviation. Normal values were verified with the Shapiro–Wilk test. For statistical analysis, we used Student’s *t* test, Tukey’s test, the ANOVA test, the Wilcoxon test, the Kruskal–Wallis test and median test. In all of the tests, the level of significance was set as p < 0.05.

## Results

The study group included 47 women (78.3%) and 13 men (21.7%). In each of the groups, the women significantly outnumbered the men. The distribution of subjects in terms of sex did not differ in a statistically significant way in the four studied groups (p = 0.329). The specific characteristics of the groups are outlined in the Table 1.

**Table 1:** Characteristics of selected features of the subjects.

Parameter	MF Group				E Group				MFE Group				C Group			
	$\overline{x}$	SD	Min	Max	$\overline{x}$	SD	Min	Max	$\overline{x}$	SD	Min	Max	$\overline{x}$	SD	Min	Max
Age (years)	35.3	7.5	25.0	47.0	30.8	8.4	22.0	49.0	29.8	7.4	20.0	46.0	34.1	8.1	20.0	49.0
Between group comparison	p = 0.189															
Body weight (kg)	69.5	8.8	57.0	70.0	71.3	16.6	50.0	70.0	70.3	15.1	49.0	65.0	69.9	8.0	55.0	70.0
Between group comparison	p = 0.981															
Body height (cm)	168.6	5.9	157.0	180.0	170.7	9.4	158.0	190.0	170.3	7.6	160.0	185.0	171.3	6.1	161.0	179.0
Between group comparison	p = 0.770															

*MF* myofascial release, *E* exercises, *MFE* myofascial release and exercises, *C* control.

As regards pain, we observed a statistically significant reduction in the intensity of pain experienced by the patients in both feet. These changes only occurred in the therapeutic intervention groups. In the pain intensity there were no statistically significant differences were observed between groups both before and after therapy (see Table 2). Because there was a significant difference in pain intensity before and after therapy in the MF, E and MFE groups, but no significant differences in pain levels were found in the comparisons between the control group and the treatment groups, the sample size was calculated to make significant differences appear. For the left foot it would be 34 people in each group, for the right foot 21 people in each group.

**Table 2:** Differences of NRS before and after therapy.

Differences of NRS before and after therapy	Group	$\overline{x}$	SD	Between measurements comparison
Left foot	MF	− 3.26	2.54	p = 0.002*
	E	− 1.93	2.12	p = 0.012*
	MFE	− 1.06	1.57	p = 0.028*
	C	− 0.80	1.69	p = 0.108
Between groups comparison	MF and E p = 0.843; MF and MFE p = 0.094; MF and C p = 0.018*; E and MFE p = 1.000; E and C p = 0.810; MFE and C p = 1.000			
Right foot	MF	− 2.66	1.63	p = 0.001*
	E	− 1.66	1.79	p = 0.012*
	MFE	− 2.06	1.96	p = 0.002*
	C	− 0.80	1.61	p = 0.068
Between groups comparison	MF and E p = 0.631; MF and MFE p = 1.000; MF and C p = 0.015*; E and MFE p = 1.000; E and C p = 0.977; MFE and C p = 0.243			

*MF* myofascial release, *E* exercises, *MFE* myofascial release and exercises, *C* control; *Statistically significant.

The greatest difference in pain intensity between before and after therapy measurements, both in the left and right foot, was observed in the MF group. The change in pain intensity in this group differed significantly compared to group C (see Table 2).

A static test of the foot load distribution showed statistically significant changes only for selected indicators. These changes concerned only the MF group and the E group only in the right foot, with more such changes recorded in the MF group. The only significant difference between the groups was observed in the Medial forefoot value in the right foot between the MF and E groups (p = 0.046) (see Tables 3 and 4).

**Table 3:** Comparison of left foot load distribution before and after therapy in static test.

	Indicators	Group	Before therapy		After therapy		Between measurements comparison
			$\overline{x}$	SD	$\overline{x}$	SD	
Left foot	Lateral forefoot	MF	10.3	5.4	12.1	4.3	p = 0.120
		E	11.4	3.5	10.6	3.8	p = 0.164
		MFE	10.9	10.3	10.2	5.9	p = 0.484
		C	14.5	3.4	13.5	3.9	p = 0.310
	Between groups comparison		MF and E p = 1.000; MF and MFE p = 1.000; MF and C p = 1.000; E and MFE p = 1.000; E and C p = 0.418; MFE and C p = 0.381				
	Medial forefoot	MF	12.5	6.1	10.9	4.9	p = 0.937
		E	9.1	4.3	10.1	6.4	p = 0.624
		MFE	11.6	7.5	11.7	7.5	p = 0.706
		C	8.5	2.7	8.0	2.8	p = 0.354
	Between groups comparison		MF and E p = 1.000; MF and MFE p = 1.000; MF and C p = 0.785; E and MFE p = 1.000; E and C p = 1.000; MFE and C p = 1.000				
	Lateral midfoot	MF	3.3	1.8	3.9	2.7	p = 0.340
		E	4.1	3.7	5.2	4.7	p = 0.224
		MFE	4.5	4.2	3.7	3.7	p = 0.169
		C	3.5	3.0	3.8	3.8	p = 0.575
	Between groups comparison		MF and E p = 1.000; MF and MFE p = 1.000; MF and C p = 1.000; E and MFE p = 1.000; E and C p = 1.000; MFE and C p = 1.000				
	Medial midfoot	MF	0.6	0.8	1.2	1.0	p = 0.310
		E	0.7	0.9	0.9	1.0	p = 0.345
		MFE	0.7	1.0	0.3	0.6	p = 0.176
		C	0.7	0.8	0.6	0.83	p = 0.715
	Between groups comparison		MF and E p = 1.000; MF and MFE p = 0.730; MF and C p = 1.000; E and MFE p = 0.809; E and C p = 1.000; MFE and C p = 1.000				
	Lateral hindfoot	MF	11.2	5.7	10.1	4.3	p = 0.346
		E	11.2	4.4	11.1	2.1	p = 0.864
		MFE	11.1	5.3	11.3	6.2	p = 0.777
		C	8.5	2.9	9.0	3.2	p = 0.690
	Between groups comparison		MF and E p = 1.000; MF and MFE p = 1.000; MF and C p = 1.000; E and MFE p = 1.000; E and C p = 0.317; MFE and C p = 0.909				
	Medial hindfoot	MF	11.1	6.3	11.2	4.0	p = 0.756
		E	14.7	3.6	14.1	4.7	p = 0.670
		MFE	13.0	7.4	10.9	6.8	p = 0.372
		C	12.5	3.9	12.6	3.5	p = 0.878
	Between groups comparison		MF and E p = 0.195; MF and MFE p = 1.000; MF and C p = 1.000; E and MFE p = 0.394; E and C p = 0.939; MFE and C p = 1.000				
	Front	MF	24.5	3.6	25.8	5.5	p = 0.593
		E	23.5	6.2	24.1	5.5	p = 0.626
		MFE	20.8	8.4	21.7	8.5	p = 0.523
		C	25.9	5.8	24.1	6.5	p = 0.208
	Between groups comparison		MF and E p = 1.000; MF and MFE p = 0.708; MF and C p = 1.000; E and MFE p = 1.000; E and C p = 1.000; MFE and C p = 1.000				
	Back	MF	27.7	6.0	25.9	3.9	p = 0.163
		E	28.1	5.9	29.2	3.1	p = 0.429
		MFE	27.8	12.2	26.9	11.6	p = 0.536
		C	23.5	6.7	24.8	5.9	p = 0.397
	Between groups comparison		MF and E p = 0.458; MF and MFE p = 1.000; MF and C p = 1.000; E and MFE p = 1.00; E and C p = 0.180; MFE and C p = 1.000				

*MF* myofascial release, *E* exercises, *MFE* myofascial release and exercises, *C* control; *Statistically significant.

**Table 4:** Comparison of right foot load distribution before and after therapy in static test.

	Indicators	Group	Before therapy		After therapy		Between measurements comparison
			$\overline{x}$	SD	$\overline{x}$	SD	
Right foot	Lateral forefoot	MF	11.2	2.8	12.7	4.2	p = 0.106
		E	11.2	3.7	10.0	2.7	p = 0.076
		MFE	10.5	4.8	10.6	5.1	p = 0.941
		C	13.3	4.4	11.8	4.3	p = 0.058
	Between groups comparison		MF and E p = 0.381; MF and MFE p = 1.000; MF and C p = 1.000; E and MFE p = 1.000; E and C p = 1.000; MFE and C p = 1.000				
	Medial forefoot	MF	9.7	3.5	11.6	3.4	p = 0.004*
		E	8.5	2.8	7.5	3.0	p = 0.101
		MFE	9.8	4.3	8.7	4.7	p = 0.243
		C	9.8	3.8	10.1	3.9	p = 0.594
	Between groups comparison		MF and E p = 0.046*; MF and MFE p = 0.363; MF and C p = 1.000; E and MFE p = 1.000; E and C p = 0.548; MFE and C p = 1.000				
	Lateral midfoot	MF	4.3	3.0	4.3	2.1	p = 0.929
		E	3.9	4.5	3.7	3.4	p = 0.441
		MFE	4.1	5.0	4.2	4.4	p = 0.575
		C	4.1	3.4	4.3	3.3	p = 0.646
	Between groups comparison		MF and E p = 1.000; MF and MFE p = 1.000; MF and C p = 1.000; E and MFE p = 1.000; E and C p = 1.000; MFE and C p = 1.000				
	Medial midfoot	MF	0.8	1.3	0.9	1.7	p = 0.933
		E	0.5	0.7	0.3	0.6	p = 0.398
		MFE	0.4	0.5	0.7	1.0	p = 0.249
		C	0.5	0.8	0.5	0.8	p = 0.735
	Between groups comparison		MF and E p = 1.000; MF and MFE p = 1.000; MF and C p = 1.000; E and MFE; p = 1.000 E and C p = 1.000; MFE and C p = 1.000				
	Lateral hindfoot	MF	7.3	2.1	6.5	2.4	p = 0.233
		E	8.5	3.8	9.5	4.0	p = 0.290
		MFE	10.3	5.2	9.6	3.9	p = 0.598
		C	8.3	2.8	7.2	2.8	p = 0.136
	Between groups comparison		MF and E p = 1.000; MF and MFE p = 0.190; MF and C p = 1.000; E and MFE p = 1.000; E and C p = 0.738; MFE and C p = 0.630				
	Medial hindfoot	MF	13.9	3.2	11.7	4.6	p = 0.152
		E	14.8	4.6	14.3	4.0	p = 0.697
		MFE	15.6	3.6	15.4	4.0	p = 0.821
		C	13.3	3.9	14.9	4.2	p = 0.091
	Between groups comparison		MF and E p = 1.000; MF and MFE p = 0.230; MF and C p = 1.000; E and MFE p = 1.000; E and C p = 1.000; MFE and C p = 0.753				
	Front	MF	22.4	4.8	25.8	5.1	p = 0.007*
		E	22.2	6.2	19.9	4.6	p = 0.042*
		MFE	22.5	10.0	24.3	10.3	p = 0.291
		C	24.6	8.1	23.3	8.4	p = 0.395
	Between groups comparison		MF and E p = 0.077; MF and MFE p = 1.000; MF and C p = 1.000; E and MFE p = 0.591; E and C p = 0.859; MFE and C p = 1.000				
	Back	MF	24.6	4.2	22.2	6.3	p = 0.211
		E	26.0	6.5	27.3	6.2	p = 0.419
		MFE	28.7	6.8	28.1	6.8	p = 0.646
		C	25.3	7.8	27.3	9.2	p = 0.192
	Between groups comparison		MF and E p = 0.548; MF and MFE p = 0.230; MF and C p = 0.958; E and MFE p = 1.000; E and C p = 1.000; MFE and C p = 1.000				

*MF* myofascial release, *E* exercises, *MFE* myofascial release and exercises, *C* control; *Statistically significant.

In the dynamic test, statistically significant changes were noted for selected indicators in both feet, in groups subjected to therapeutic intervention. Similarly to the static test, in the dynamic test the most indicators changed in a statistically significant way in the MF group. In between groups comparison no statistically significant differences were observed in both feet (see Tables 5 and 6).

**Table 5:** Selected indicators of left foot load and ground reaction forces in the dynamic test.

	Indicators	Group	Before therapy		After therapy		Between measurements comparison
			$\overline{x}$	SD	$\overline{x}$	SD	
Left foot	Area (cm^2^)	MF	119.1	14.4	111.5	14.6	p = 0.001*
		E	124.1	17.7	121.1	20.1	p = 0.105
		MFE	110.9	20.0	115.2	16.5	p = 0.104
		C	118.9	13.9	119.9	13.7	p = 0.359
	Between groups comparison		MF and E p = 0.976; MF and MFE p = 1.000; MF and C p = 0.809; E and MFE p = 1.000; E and C p = 1.000; MFE and C p = 1.000				
	Stance time (ms)	MF	691.5	116.5	703.5	93.5	p = 0.645
		E	725.5	149.7	692.6	85.6	p = 0.629
		MFE	713.1	98.4	749.7	81.1	p = 0.065
		C	706.9	72.1	716.7	67.7	p = 0.549
	Between groups comparison		MF and E p = 1.000; MF and MFE p = 0.630; MF and C p = 1.000; E and MFE p = 0.210; E and C p = 1.000; MFE and C p = 1.000				
	vGRF F1 (N)	MF	556.8	92.3	561.4	102.3	p = 0.816
		E	602.0	149.2	564.8	124.5	p = 0.080
		MFE	550.8	126.7	566.8	113.7	p = 0.598
		C	557.5	80.4	536.4	103.6	p = 0.348
	Between groups comparison		MF and E p = 1.000; MF and MFE p = 1.000; MF and C p = 1.000; E and MFE p = 1.000; E and C p = 1.000; MFE and C p = 1.000				
	vGRF F2 (N)	MF	394.8	69.8	388.9	64.2	p = 0.531
		E	387.1	145.7	379.7	126.7	p = 0.875
		MFE	384.5	103.3	384.2	103.0	p = 0.954
		C	385.8	79.9	392.3	77.0	p = 0.532
	Between groups comparison		MF and E p = 1.000; MF and MFE p = 1.000; MF and C p = 1.000; E and MFE p = 1.000; E and C p = 1.000; MFE and C p = 1.000				
	vGRF F3 (N)	MF	667.8	86.7	650.9	99.3	p = 0.081
		E	679.6	170.3	689.2	162.8	p = 0.041*
		MFE	663.9	158.5	668.5	154.6	p = 0.251
		C	662.4	84.4	669.9	87.7	p = 0.219
	Between groups comparison		MF and E p = 1.000; MF and MFE p = 1.000; MF and C p = 1.000; E and MFE p = 1.000; E and C p = 1.000; MFE and C p = 1.000				

*MF* myofascial release, *E* exercises, *MFE* myofascial release and exercises, *C* control; *Statistically significant.

**Table 6:** Selected indicators of right foot load and ground reaction forces in the dynamic test.

	Indicators	Group	Before therapy		After therapy		Between measurements comparison
			$\overline{x}$	SD	$\overline{x}$	SD	
Right foot	Area (cm^2^)	MF	120.7	13.3	114.3	14.2	p = 0.014*
		E	122.1	19.3	119.2	18.0	p = 0.086
		MFE	916.7	110.8	887.6	122.9	p = 0.346
		C	122.0	15.1	123.1	14.7	p = 0.547
	Between groups comparison		MF and E p = 1.000; MF and MFE p = 1.000; MF and C p = 0.859; E and MFE p = 1.000; E and C p = 1.000; MFE and C p = 0.842				
	Stance time (ms)	MF	695.3	83.8	701.1	99.9	p = 0.761
		E	719.2	108.4	688.3	75.8	p = 0.280
		MFE	710.0	97.1	759.5	85.6	p = 0.019*
		C	714.1	76.0	714.7	73.4	p = 0.961
	Between groups comparison		MF and E p = 1.000; MF and MFE p = 0.296; MF and C p = 1.000; E and MFE p = 0.116; E and C p = 1.000; MFE and C p = 0.995				
	vGRF F1 (N)	MF	565.2	100.9	568.6	100.0	p = 0.896
		E	581.3	136.2	560.0	149.8	p = 0.339
		MFE	535.6	109.2	551.6	124.1	p = 0.520
		C	585.5	83.8	578.0	95.8	p = 0.670
	Between groups comparison		MF and E p = 1.000; MF and MFE p = 1.000; MF and C p = 1.000; E and MFE p = 1.000; E and C p = 1.000; MFE and C p = 1.000				
	vGRF F2 (N)	MF	397.6	89.1	401.3	94.1	p = 0.407
		E	393.5	152.6	379.6	110.1	p = 0.729
		MFE	392.2	89.0	392.2	89.0	p = 0.000*
		C	381.4	67.6	409.5	69.9	p = 0.052
	Between groups comparison		MF and E p = 1.000; MF and MFE p = 1.000; MF and C p = 1.000; E and MFE p = 1.000; E and C p = 0.708; MFE and C p = 1.000				
	vGRF F3 (N)	MF	658.1	95.8	663.0	93.2	p = 0.500
		E	677.5	169.7	678.9	157.5	p = 0.795
		MFE	675.8	147.5	674.2	149.4	p = 0.653
		C	646.8	112.7	673.1	76.8	p = 0.133
	Between groups comparison		MF and E p = 1.000; MF and MFE p = 1.000; MF and C p = 1.000; E and MFE p = 1.000; E and C p = 1.000; MFE and C p = 1.000				

*MF* myofascial release, *E* exercises, *MFE* myofascial release and exercises, *C* control; *Statistically significant.

In the dynamic test assessing the support phase of the foot, statistically significant changes in before and after therapy measurements comparison were observed for all the subphases. Far more statistically significant changes were found for the right foot and for the MFE group. There were no statistically significant changes in the between group comparison for both feet (see Tables 7 and 8).

**Table 7:** Selected indicators of left foot loading during the support phase in the dynamic test.

	Indicators	Group	Before therapy		After therapy		Between measurements comparison
			$\overline{x}$	SD	$\overline{x}$	SD	
Left foot	LR duration (ms)	MF	93.3	17.6	95.2	12.1	p = 0.550
		E	97.9	19.4	96.5	8.7	p = 0.801
		MFE	97.9	14.5	100.2	12.4	p = 0.394
		C	95.8	12.7	97.1	10.7	p = 0.625
	Between groups comparison		MF and E p = 1.000; MF and MFE p = 0.723; MF and C p = 1.000; E and MFE p = 1.000; E and C p = 1.000; MFE and C p = 1.000				
	LR maxload (%)	MF	51.3	9.9	51.6	12.2	p = 0.929
		E	57.1	12.7	57.1	12.7	p = 1.000
		MFE	47.0	11.1	49.6	8.5	p = 0.286
		C	53.3	6.1	52.5	10.3	p = 0.775
	Between groups comparison		MF and E p = 1.000; MF and MFE p = 1.000; MF and C p = 1.000; E and MFE p = 0.423; E and C p = 1.000; MFE and C p = 1.000				
	MidSt duration (ms)	MF	231.5	39.1	233.4	31.2	p = 0.816
		E	239.1	49.5	221.9	28.4	p = 0.244
		MFE	232.3	35.4	248.0	30.3	p = 0.044*
		C	233.3	20.4	238.3	31.6	p = 0.464
	Between groups comparison		MF and E p = 1.000; MF and MFE p = 1.000; MF and C p = 1.000; E and MFE p = 0.135; E and C p = 0.958; MFE and C p = 1.000				
	MidSt maxload (%)	MF	91.1	9.0	91.2	8.4	p = 0.694
		E	85.8	10.7	82.4	11.2	p = 0.157
		MFE	82.4	11.8	85.5	9.3	p = 0.347
		C	91.3	9.3	92.1	9.9	p = 0.753
	Between groups comparison		MF and E p = 0.230; MF and MFE p = 1.000; MF and C p = 1.000; E and MFE p = 1.000; E and C p = 0.107; MFE and C p = 0.604				
	TSt duration (ms)	MF	225.5	39.3	232.7	33.3	p = 0.439
		E	239.5	49.7	230.9	28.4	p = 0.733
		MFE	236.3	32.8	250.3	24.4	p = 0.074
		C	233.1	25.9	235.9	23.3	p = 0.629
	Between groups comparison		MF and E p = 1.000; MF and MFE p = 0.326; MF and C p = 1.000; E and MFE p = 0.153; E and C p = 1.000; MFE and C p = 0.885				
	TSt maxload (%)	MF	98.7	3.8	98.3	3.8	p = 0.715
		E	99.0	3.5	99.2	2.1	p = 1.000
		MFE	98.7	3.0	99.9	0.3	p = 0.074
		C	98.9	2.6	98.7	2.7	p = 0.583
	Between groups comparison		MF and E p = 1.000; MF and MFE p = 1.000; MF and C p = 1.000; E and MFE p = 1.000; E and C p = 1.000; MFE and C p = 1.000				

*MF* myofascial release, *E* exercises, *MFE* myofascial release and exercises, *C* control; *Statistically significant.

**Table 8:** Selected indicators of right foot loading during the support phase in the dynamic test.

	Indicators	Group	Before therapy		After therapy		Between measurements comparison
			$\overline{x}$	SD	$\overline{x}$	SD	
Right foot	LR duration (ms)	MF	95.1	16.5	96.5	15.9	p = 0.702
		E	99.0	15.3	94.9	10.0	p = 0.157
		MFE	94.2	14.4	102.6	13.9	p = 0.009*
		C	97.0	11.0	98.3	13.3	p = 0.656
	Between groups comparison		MF and E p = 1.000; MF and MFE p = 1.000; MF and C p = 1.000; E and MFE p = 1.000;E and C p = 1.000; MFE and C p = 1.000				
	LR maxload (%)	MF	55.3	9.6	51.4	10.5	p = 0.286
		E	57.6	12.5	54.8	6.7	p = 0.880
		MFE	43.9	10.5	45.5	12.4	p = 0.508
		C	56.9	16.5	53.8	9.7	p = 0.306
	Between groups comparison		MF and E p = 1.000; MF and MFE p = 0.715; MF and C p = 1.000; E and MFE p = 0.071; E and C p = 1.000; MFE and C p = 0.227				
	MidSt duration (ms)	MF	230.3	24.8	234.4	33.8	p = 0.038*
		E	236.7	35.7	228.3	25.0	p = 0.974
		MFE	236.4	33.5	249.9	29.8	p = 0.046*
		C	234.0	27.1	236.7	27.8	p = 0.605
	Between groups comparison		MF and E p = 1.000; MF and MFE p = 0.809; MF and C p = 1.000; E and MFE p = 0.208; E and C p = 1.000; MFE and C p = 1.000				
	MidSt maxload (%)	MF	91.5	7.5	91.1	10.2	p = 0.826
		E	89.1	7.9	84.6	7.5	p = 0.012*
		MFE	80.5	10.6	84.1	12.7	p = 0.103
		C	81.1	15.4	91.1	11.5	p = 0.701
	Between groups comparison		MF and E p = 1.000; MF and MFE p = 0.474; MF and C p = 1.000; E and MFE p = 1.000; E and C p = 0.746; MFE and C p = 0.665				
	TSt duration (ms)	MF	232.2	27.2	228.5	33.5	p = 0.519
		E	238.3	37.1	227.2	29.3	p = 0.061
		MFE	220.7	62.4	250.7	29.3	p = 0.041*
		C	239.9	28.7	238.5	24.3	p = 0.828
	Between groups comparison		MF and E p = 1.000; MF and MFE p = 0.268; MF and C p = 1.000; E and MFE p = 1.000; E and C p = 1.000; MFE and C p = 1.000				
	TSt maxload (%)	MF	99.0	2.7	98.4	2.4	p = 0.554
		E	98.4	3.3	99.3	2.6	p = 0.144
		MFE	100.0	0.0	99.5	1.0	p = 0.103
		C	99.1	2.1	97.4	5.3	p = 0.248
	Between groups comparison		MF and E p = 0.817; MF and MFE p = 1.000; MF and C p = 1.000; E and MFE p = 1.000; E and C p = 1.000; MFE and C p = 1.000				

*MF* myofascial release, *E* exercises, *MFE* myofascial release and exercises, *C* control; *Statistically significant.

## Discussion

Although the impact of various therapies on pain is broadly discussed in scientific research, there are very few reports in the available literature on the effectiveness of myofascial release in decreasing pain in the foot, especially in flat feet. Kuhar et al.^36^ examined people with pain in a properly arched foot, in whom they used myofascial techniques in combination with exercises, foot baths and ultrasounds. After 10 days of therapy, the authors obtained a significant reduction of pain in the subjects. Pant et al.^37^ assessed correctly arched feet in patients with plantar fasciitis. They used myofascial release (group A) and static relaxation techniques (group B). The results revealed similar effectiveness of both techniques in reducing pain with a slight advantage of myofascial release. Cleland et al.^38^ compared the effects of two 4-week rehabilitation programs on reducing heel pain. The first program consisted of ionophoresis with exercises, while the second one involved mobilization of the joints of the lower extremities, myofascial release and exercises. The findings demonstrated better results in the treatment of heel pain when using the program with myofascial release. Yadav et al.^24^ compared the therapeutic effect between ultrasound procedures and myofascial therapy in people with plantar fasciitis. Their results support greater effectiveness of manual therapy. Harlapur et al.^25^ also studied people suffering from plantar fasciitis who had both myofascial release and positional relaxation techniques done. Based on their findings, they conclude that both methods proved to be equally effective in the treatment of that condition. In our own research, we assess the use of myofascial release in people with low-arch foot pain. A considerable alleviation of pain was found in all treatment groups, with the greatest improvement noted in the group undergoing only myofascial release (MF). In group C (control) no changes were observed. In the between group comparisons, statistically significant differences were observed only between the MF group and the C group. No statistically significant differences were observed between the other groups (MFE and E) and the C group. This indicates a greater ability of myofascial techniques to reduce pain in the form of individual therapy compared to combined therapies or other techniques. In foot diagnostics, the foot pressure test on the ground is more and more commonly used, but there are still no established norms of pressure value and its distribution^39^. A major number of the existing publications on the problem of static foot load concern the effect of arching/structure of the foot on the obtained pressure values^5,40^, however, there are no reports on the influence of physiotherapy techniques on the distribution of foot load in people with flat foot pain. Martinez-Jimenez et al.^41^ investigated the effectiveness of myofascial release in 20 healthy people. The therapeutic intervention lasted five minutes, and the utility of the procedure was evaluated on a sensory platform during a balance test with eyes open and closed. The results of this study have shown the impact of the myofascial release on increasing the loading surface and pressure in the forefoot. Our research only partially confirms the literature reports, which may be related to the longer duration of the intervention and other evaluation methods. In our research, patients underwent 4 weeks of therapeutic treatment. Each tested foot was divided into eight loading regions: two forefoot, two midfoot and two hindfoot (lateral and medial) regions, respectively, and two regions for the entire foot (front and back). The therapeutic techniques applied for over 4 weeks revealed an effect on changes in foot loading in individual regions, but only in some groups and only for the right foot. Statistically significant changes occurred in the medial forefoot and front of the foot for the MF group and the front of the foot for the E group, so the myofascial release was shown to have a slightly greater impact on loading changes within the feet. In group C (control), no changes were recorded. However, the between group comparisons did not indicate a particularly strong influence of the myofascial release techniques on the selected static foot indicators.

In an the overwhelming number of publications that study foot pressure on the ground, such measurements are carried out under dynamic conditions. However, they consider factors such as foot structure, weight, sex, age or range of motion^1,42,43^ while rarely evaluating the impact of therapeutic intervention on dynamic foot indicators. Among these recent reports, there are no publications on the impact of myofascial release on the above indicators.

Panichawit et al.^44^ examined five people with lowered arching of the foot, subjecting them to an 8-week therapeutic program, consisting of stretching exercises for selected calf muscles and of exercises strengthening selected foot muscles. The authors noted a reduction in contact area within the big toe, the metatarsal head and the middle part of the foot in the studied persons. Taspinar et al.^45^ investigated the effects of using insoles, footwear modifications and exercises using a platform testing foot pressure on the ground. The therapeutic intervention lasted 4 weeks and the authors did not observe any statistically significant changes in foot pressure, either directly after or 3 months after the end of treatment. Boozarii et al.^46^ focused on examining changes in the vGRF index under the influence of a motor task in 17 people with flat feet compared to people with properly arched feet. Before performing the motor task, people with flat foot obtained higher values of this indicator compared to people with normal foot arches. After the test, the difference between the groups increased. Our research confirms Tespinar’s reports^45^. In our study, an assessment of foot pressure while walking was performed in 60 subjects before and after therapy (4 weeks). Statistically significant differences were obtained only for a few indicators in the groups in which therapeutic interventions were used. For the MFE group, the right foot stance time increased significantly, and in group E, the vGRF F3 index in the left foot changed. In the MF group, the loading area for both feet changed significantly. In group C (control) no changes were observed. Additionally, no statistically significant changes were observed in the comparisons between groups for both feet.

Available reports on the effectiveness of various physiotherapeutic techniques on dynamic foot indicators do not analyze the impact of applied therapies on gait phases in relation to flat feet with pain. However, such an analysis seems to be warranted, because, as it has already been mentioned, incorrect arching of the foot and related pain may affect the biomechanics of gait by disturbing the pressure of the feet on the ground^2,47^. In our research, we assessed the effectiveness of the proposed 4-week therapies. The most statistically significant changes were observed in the MFE group, especially for the right foot, where the duration (ms) for all three subphases of the support phase of gait changed. In the same group, in the left foot, the change was noted only for the duration of the MidStance subphase. In the other treatment groups, the statistically significant changes occurred only in the left foot MidStance subphase, in terms of duration and maximum load for the MF group and E group, respectively. No changes were observed in the C (control) group.

The subject of the effectiveness of myofascial therapy for flat foot disorder seems to remain an important point of investigation. The results of our research, as well as the relatively small amount of available publications on this subject indicate the need for further research in this direction.

## Conclusion

Our study shows that both exercise and exercise combined with myofascial release techniques, and especially myofascial release techniques alone, significantly reduce pain in a flat foot. Our study, as one of the first describing the effect of various types of therapy, shows a limited influence of both exercises and myofascial release techniques on selected static and dynamic indicators of a flat foot.

## Study limitation

This study is not without limitations. An important aspect that could affect trial results is a relatively small number of people in the study groups. This sample size could be a cause of e.g. the potential lack of power in differentiating between groups in pre and posttest comparisons for many parameters.
